# Management of Axillary Contracture in Poland Syndrome: Differentiating Fibrous Band and Skin for Optimal Release

**DOI:** 10.3390/jcm12154957

**Published:** 2023-07-28

**Authors:** Rikako Matsuura, Yusuke Shimizu, Naoki Matsuura, Edward Hosea Ntege, Naoki Wada

**Affiliations:** 1 Department of Plastic and Reconstructive Surgery, Graduate School of Medicine, University of the Ryukyus, Okinawa 903-0215, Japan; nodaridano@gmail.com (R.M.); prsmatsuura@gmail.com (N.M.); ehntege@gmail.com (E.H.N.); 2 Department of Pathology and Oncology, Graduate School of Medicine, University of the Ryukyus, Okinawa 903-0215, Japan; wadan@med.u-ryukyu.ac.jp

**Keywords:** Poland syndrome, axillary contracture, fibrous band, Z-plasty, tendon tissue, case report

## Abstract

Poland syndrome (PS), an uncommon congenital unilateral aplasia of chest wall muscles, may exhibit rare accompanying signs, such as axillary webbing or contractures. The existing literature on the specific management of axillary contractures is limited. In this report, we present the case of a 10-year-old girl with PS manifesting an axillary web containing a fibrous band, which was successfully surgically corrected by a double-opposing Z-plasty. Our surgical approach entailed a meticulous distinction between the deep fibrous band and the superficial cutaneous layer, guided by histopathological findings that indicated the presence of tendon-like tissue, ultimately yielding excellent outcomes. This report will help expand knowledge by highlighting the unique manifestation of PS and emphasizing the importance of employing appropriate treatment approaches. Moreover, addressing both tendon and skin components is essential for optimal contracture release in PS.

## 1. Introduction

Poland syndrome (PS), an uncommon congenital condition, is characterized by the absence or underdevelopment of the pectoralis major muscle, accompanied with anomalies such as hand abnormalities, rib defects, and breast hypoplasia [1,2,3,4,5,6]. In 1841, Alfred Poland first described PS based on an autopsy [4]. Cases similar to PS were documented in the early 19th century, and Patrick Clarkson coined the term “Poland’s syndactyly” [5,7,8,9,10,11,12]. Despite some controversies, Poland’s contributions remain significant in understanding and classifying PS [8,13]. PS occurs in approximately 1 in 20,000–50,000 live births, with variations reported across populations [14]. PS is more commonly observed in men than in women, with a male-to-female ratio ranging from 2:1 to 3:1 [15]. In addition, PS is more prevalent on the right side of the body, accounting for approximately 70–75% of cases [15].

The etiology of PS is still debatable, and the vascular disruption theory is the most widely accepted mechanism [16]. This theory states that the disruption of the proximal subclavian artery and its branches during the 6th week of gestation leads to deficient blood flow, resulting in regional tissue loss [16,17]. This disruption in embryonic vessels that caused vascular disruption was referred to as the subclavian artery supply disruption sequence [16]. Intrinsic factors, such as thrombi and emboli, and external mechanical factors, such as cervical ribs, aberrant muscles, and amniotic bands, can contribute to the interruption or reduction of blood flow to the subclavian artery and its branches [2,18,19,20,21]. Maternal factors, such as smoking and cocaine abuse, were also suggested as potential risk factors [21]. Genetic and extrinsic factors may also interfere with the migration of the pectoralis major muscle and digital separation during embryogenesis [22,23]. Familial cases of PS have been reported, suggesting a possible autosomal dominant inheritance with variable penetrance [24,25,26]. Recent studies have identified de novo mutations and duplications in specific chromosomal regions, which may contribute to PS development [23,27]. PS can also coexist with other syndromes, such as Moebius syndrome, Klippel–Feil syndrome, and Pierre–Robin sequence [16,28].

The clinical spectrum of PS involves the unilateral absence or hypoplasia of the pectoralis major muscle, typically affecting the sternal and costal portions [4,5]. PS exhibits varying severities of muscle involvement, accompanied by breast hypoplasia and chest wall deformities. Pubertal breast asymmetry unveils the anomaly, requiring reconstruction of the nipple–areolar complex because of variations [29,30]. In addition, PS can present with various upper limb abnormalities, such as syndactyly, symbrachydactyly, brachydactyly, hypoplasia or aplasia of the hand or fingers, and clinodactyly [31,32]. Thoracic anomalies in PS may involve the unilateral absence or fusion of ribs, which is often associated with scoliosis or chest wall asymmetry [31,32]. Other associated anomalies reported in some PS cases include lung herniation, dextrocardia, eventration of the diaphragm, vertebral anomalies, and urogenital abnormalities [33,34,35]. Notably, studies have also reported cutaneous webbing in areas such as the pectoral and axillary regions [31,32].

PS management is multifaceted, and treatment approaches may vary depending on the manifestations [36,37,38,39]. Despite the current extensive understanding of the disease, only a few studies have addressed the presence of axillary contractures. PS-associated axillary contractures may occur because of a rudimentary pectoralis muscle originating from the sternum, or they may manifest as cutaneous webbing containing fibrous bands that restrict mobility and cause functional impairment [39]. Thus, deciphering unique manifestations and identifying precise treatment approaches for PS are crucial for expanding knowledge. This report focuses on achieving the optimal release of PS-associated axillary contractures induced by interwoven connective tissues, specifically fibrous bands. This approach involves precise histopathological differentiation and a double-opposing Z-plasty in a 10-year-old patient, supported by a comprehensive literature review.

## 2. Case Description

**Figure 1:**
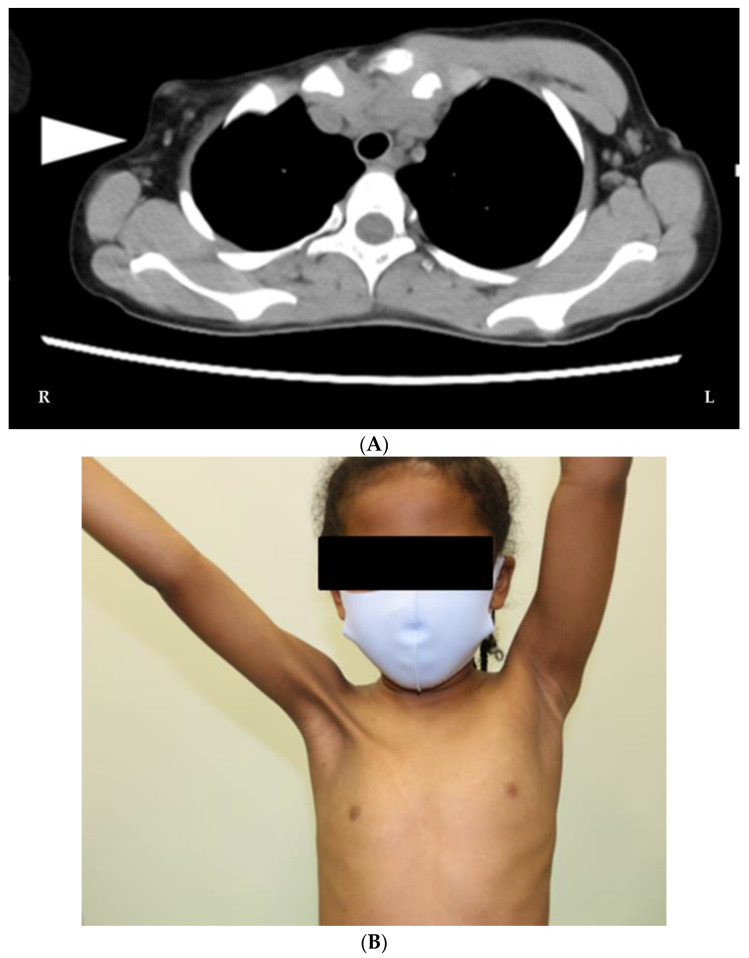
(**A**) Chest CT scan of a 10-year-old girl with axillary contractures revealed the absence of pectoral muscles in the right thoracic wall (white arrowhead). (**B**) Preoperative photograph of a 10-year-old girl with PS and axillary contractures demonstrating limited range of motion of the right shoulder joint.

A 10-year-old girl with persistent complaints of axillary webbing and restricted movements of the right shoulder joint for over 2 years was referred to our Department of Plastic and Reconstructive Surgery for further evaluation and surgical management. She had been diagnosed with PS by a primary physician and had no significant family or medical history. The assessment by the primary physician involved a radiological examination, wherein computed tomography (CT) was performed to evaluate the underlying anatomical abnormalities. CT revealed hypoplasia of the right pectoralis major muscle, confirming the presence of muscular asymmetry and structural anomalies. Imaging studies also provided detailed information regarding the integrity of adjacent structures, such as the ribs, sternum, and vertebrae, ruling out any associated abnormalities or skeletal malformations (Figure 1A).

Upon further evaluation, the patient appeared slightly apprehensive but was in overall good condition, having no signs of pallor, edema, cyanosis, jaundice, cervical lymphadenopathy, or generalized lymphadenopathy. Systemic examinations yielded normal results. An axillary contracture with limited range of motion (ROM) in the right shoulder joint, specifically in abduction and flexion (Figure 1B), was observed. The functional limitations of the shoulder joint significantly affected the patient’s daily living activities and necessitated surgical intervention to improve her quality of life.

**Figure 2:**
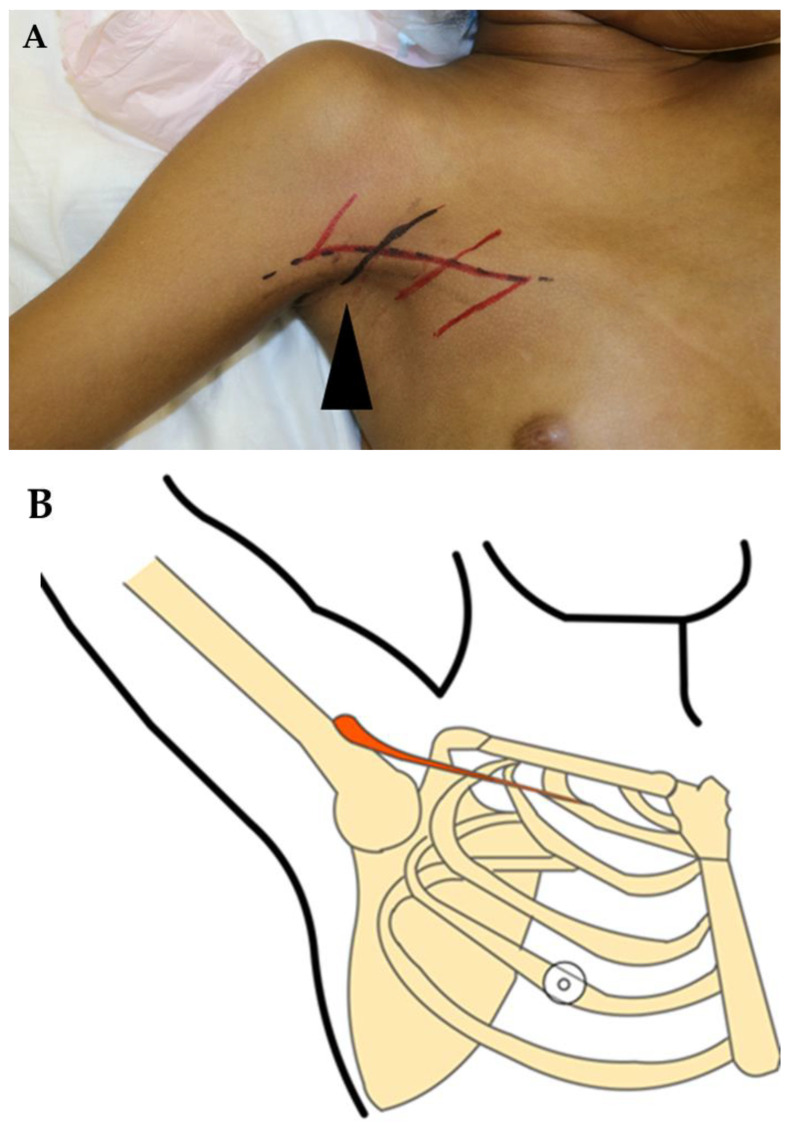
(**A**) Preoperative illustrative skin markings for the Z-plasty plan at the right shoulder joint (black arrowhead). The Z-plasty design involved elevating the right arm and making an excision along the black line. (**B**) Illustrative diagram showing the right-side axillary contracture.

The surgical treatment was performed under general anesthesia, with careful consideration of the patient’s unique anatomical presentation and functional limitations. The right shoulder joint was positioned in abduction and flexion, creating the necessary tension for the three Z-plasty formations. These Z-plasty formations aimed to release the contracture and restore optimal functionality while minimizing scar formation and maximizing esthetic outcomes (Figure 2A,B).

**Figure 3:**
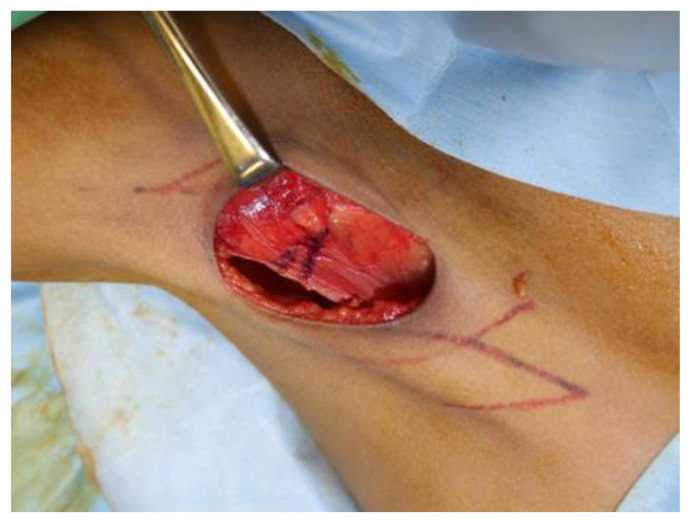
Intraoperative photograph of the 10-year-old girl with PS and axillary contracture, indicating a wedge-shaped excision of the fibrous band.

Following the initial skin incision, a deeper incision was made through the deep fascia, allowing for the precise dissection of the fibrous band that caused the contracture. The fibrous band was the primary cause of the limited ROM, and a wedge-shaped resection of the central portion with the highest tension was performed to ensure complete release (Figure 3). Two of the initial three Z-plasty designs were successfully executed. To optimize wound drainage and minimize postoperative complications, the J-VAC™ Drainage System (Ethicon, Inc., Somerville, NJ, USA), including a 10-Fr. BLAKE™ Silicon Drain (Ethicon, Inc.), was subcutaneously placed and connected to the J-VAC™ suction reservoir. Intraoperatively, the complete release of the axillary contracture was confirmed, ensuring optimal functional outcomes (Supplementary Video S1).

**Figure 4:**
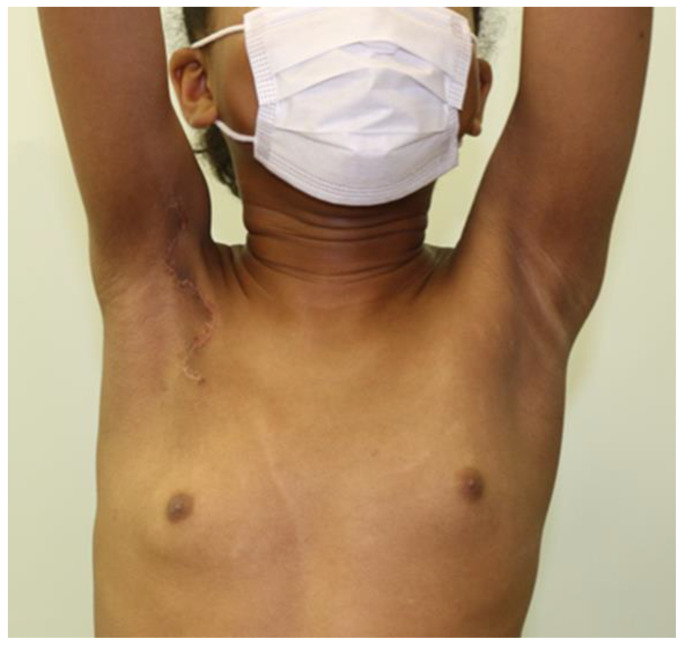
Postoperative photograph of the 10-year-old girl with PS and axillary contracture 5 months after surgery, indicating increased range of motion of the right shoulder joint.

Postoperatively, the patient was discharged on day 6, and a comprehensive rehabilitation program was initiated, focusing on restoring the ROM in the right shoulder joint. Diligent rehabilitation efforts, combined with the successful surgical treatment, have yielded positive results. At the 5-month follow-up, the patient continues to demonstrate remarkable progress without any signs of recontraction (Figure 4). These outcomes highlight the effectiveness of the surgical approach and emphasize the importance of tailored postoperative care and rehabilitation in achieving favorable patient outcomes.

**Figure 5:**
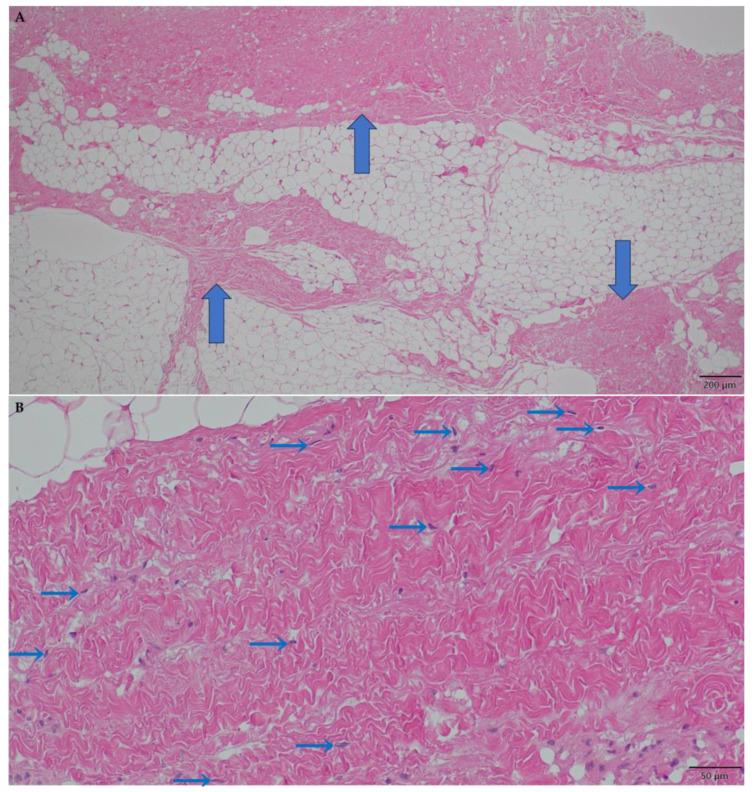

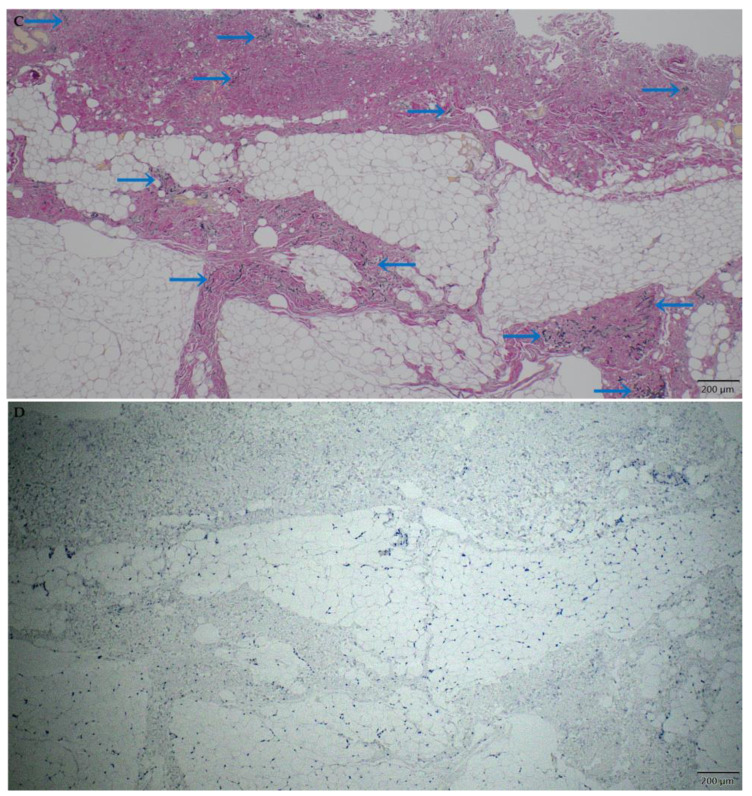
Histopathological image of the excised axillary tissue contracture. (**A**) Hematoxylin and eosin (H&E) staining image showing the fibers (wide blue arrows) in fatty component (H&E staining: objective, ×4; scale bar, 200 μm). (**B**) The fibers consisted mainly of dense collagen fibers, indicating a tendon-like nature. Scattered nuclei (narrow blue arrows) of fibroblasts are shown (H&E staining: objective, ×20; scale bar, 50 μm). (**C**) Elastica van Gieson (EVG) staining image revealed a predominantly dense collagen fiber composition (red staining) with limited presence of elastic fibers. EVG staining image highlighted fewer elastic fibers (black staining indicated by the narrow blue arrows) than collagen fibers (red staining) (EVG staining: objective, ×4; scale bar, 200 μm). (**D**) Immunohistochemical staining image showing the absence of myoglobin-positive skeletal muscles. No myoglobin-positive skeletal muscles were found in the excised specimen (myoglobin immunohistochemistry: objective, ×4; scale bar, 200 μm).

To deeply understand the histological characteristics of the axillary tissue contracture, a histopathologic examination was conducted. The excised axillary contracture tissue was fixed in a 10% neutral buffered formalin solution, embedded in paraffin, and cut into 3-μm-thick sections. These sections underwent hematoxylin and eosin (H&E), Elastica van Gieson (EVG), and immunohistochemical staining. All slides were dewaxed in xylene, dehydrated in graded alcohol, and rehydrated in distilled water. H&E staining was performed using Mayer’s Hematoxylin Solution (Product no. 30002, Muto Pure Chemicals Co., Ltd., Tokyo, Japan) and Eosin Solution (Product no. 32042, Muto Pure Chemicals Co., Ltd.). EVG staining was performed using Maeda’s Resorcin-Fuchsin Solution (Product no. 4032, Muto Pure Chemicals Co.), Van Gieson’s Stain Solution A (Product no. 4036, Muto Pure Chemicals Co., Ltd.), and Van Gieson’s Stain Solution B (Product no. 4037, Muto Pure Chemicals Co., Ltd.). Immunohistochemical staining was performed using an automated staining system (Dako Autostainer Link 48, Agilent/Dako, Santa Clara, CA, USA). For immunohistochemistry, monoclonal mouse anti-human myoglobin antibody (clone MYO18, dilution 1:500, Novocastra) was used. This comprehensive histopathologic examination revealed a predominantly dense collagen fiber composition with a limited presence of elastic fibers, confirming the tendon-like nature of the fibrous band (Figure 5A–C). No myoglobin-positive skeletal muscles were found in the excised specimen (Figure 5D). These findings provided crucial insights into the pathogenesis and nature of the axillary contracture, contributing to the overall understanding of the patient’s condition.

### Literature Review: Literature Search Strategy

The literature review focused primarily on original English articles concerning patients with PS. Non-English articles were assessed for relevance and the presence of new information. Medline, Embase, and Web of Science were thoroughly searched using various combinations of the following search terms: PS, axillary contractures, fibrous band, Z-plasty, surgical management, and dermatology. No limitations on the publication date were implemented, and the earliest article included was published in 1841.

The articles were manually screened and selected based on the inclusion and exclusion criteria in a two-step process conducted by two authors (R.M. and E.H.N.). In the case of discrepancies between the authors’ selections, a third author (N.M. or Y.S.) acted as an arbitrator to reach a consensus. The title and abstract of the selected articles were reviewed, followed by full-text screening. Table 1 summarizes the findings of the literature search.

## 3. Discussion

PS is a rare congenital condition originally described by Alfred Poland. It is characterized by the underdevelopment or absence of chest muscles on one side of the body, typically the right side [3,15]. In addition to musculoskeletal abnormalities, PS can present with various dermatological manifestations [31,32]. These manifestations encompass hypoplasia or absence of the breast, nipple and areola hypoplasia, cutaneous webbing (syndactyly) between the fingers, exceptionally rare syndactyly of the toes, hyperhidrosis, and axillary contractures. Axillary contractures, also known as axillary web syndrome or cording, are relatively uncommon complications of PS [1,2,4,5,6]. Axillary contractures involve the formation of tight bands of tissue in the axilla, leading to pain and restricted ROM in the affected arm. These contractures can also occur in individuals who have experienced extensive burns, undergone surgery, or received radiation therapy in the axillary region. Despite the rare occurrence of axillary contractures in PS, treatment approaches vary depending on the needs of each patient. Some cases may not require treatment, particularly if the cosmetic differences are minor and do not hinder functionality. However, some patients face physical challenges, such as ROM limitations and chest wall deformities, causing esthetic concerns. These physical manifestations contribute to psychological distress, such as self-consciousness, low self-esteem, and anxiety. Noticeable asymmetry and variations in breast size and shape affect social interactions and psychological wellbeing. Surgical interventions, such as breast reconstruction, can address these issues [29,30]. In addition to physical therapy, psychosocial rehabilitation may be recommended to address PS-related functional limitations, including associated axillary contractures. In the present case, a 10-year-old girl with PS presented with axillary contractures, and positive outcomes were achieved through precise surgical intervention and physiotherapy. By differentiating between the deep axillary fibrous band and the superficial skin, the contracture was successfully treated. This approach ensures the appropriate release of the contracture, contributing to optimal results [39,40,41].

Only three documented cases of axillary contracture in PS were reported in the literature [39,40,41]. As shown in Table 1, case 2 highlights the surgical procedure performed on a 6-year-old girl. The intervention involved the excision of a fibrous band using a step-ladder technique, followed by skin removal through a double-opposing Z-plasty to alleviate the scar contracture [39]. Furthermore, case 3 involved a 9-month-old girl who underwent fibrous band excision and a single Z-plasty [41]. In the present case, both the deep fibrous band and the skin were addressed. The wedge-shaped technique was employed to resect the central region of the fibrous band, which exhibited the highest tension. Unlike the step-ladder approach, a simple wedge resection was sufficient in our case. In addition, we performed two Z-plasties to effectively release the skin contracture [39,40,41].

Z-plasty is widely utilized in plastic surgery for various purposes, such as scar lengthening, scar concealment, or scar realignment. Some common variations of basic Z-plasty include double-opposing, unequal-triangle, 4-flap, compound, and planimetric Z-plasties [42]. Axillary contracture revision surgery is commonly employed for postburn scars, and various techniques, such as Z-plasty, square-flap, 4-flap, and 5-flap Z-plasties, are utilized based on the severity of the contracture [43,44]. The choice of the technique depends on the needs of the patient. The 4- and 5-flap techniques are preferred for strong contractures owing to their high success rates, whereas Z-plasty with minimal scarring is suitable for milder cases [43,44,45].

In our case, the pathology results revealed that the fibrous band consisted of tendon-like tissue [39]. This finding corresponds to a previous report of myosclerotic changes observed in case 3 [39]. In addition, two patients who underwent surgery reported the presence of residual pectoralis major muscle attached to the anterior thoracic region [39,40]. Similarly, in the present case, the fibrous band was presumed to be tendinous tissue originating from the residual pectoralis major muscle, as it was attached to the second rib [39,40]. These findings suggest that deep tendon-like structures, along with the skin, may contribute to axillary contractures in PS. The identified fibrous band attachment to the second rib could have implications for future breast development. Long-term follow-up is recommended to monitor and plan for appropriate breast reconstruction, considering the axillary web and potential future pathological developments.

In conclusion, this case highlights the successful management of axillary contractures in a 10-year-old girl with PS. By differentiating between the fibrous band and the skin, precise surgical interventions were performed, leading to favorable outcomes, albeit with a long-term follow-up recommendation. The distinction between the tendon and skin involvement is crucial in achieving optimal contracture release. This case contributes to the understanding of axillary contractures in PS and emphasizes the importance of tailored treatment strategies that address the specific anatomical factors associated with PS.

## Figures and Tables

**Table 1:** Three currently reported cases of axillary contractures.

Case	Sex	Body Side	Age at Diagnosis	Age at Surgery	Surgical Technique	Reference
1	Female	Right	Neonate	Pending		[40]
2	Female	Left	6 years	6 years	Double-opposing Z-plasty plus step-ladder incision-lengthening	[39]
3	Female	Right	3 months	9 months	Z-plasty	[41]

## Data Availability

The datasets used during the current study are available from the corresponding author on reasonable request.

## Supplementary Materials

The following supporting information can be downloaded at: https://www.mdpi.com/article/10.3390/jcm12154957/s1, Video S1: Demonstration of surgical release for PS-associated axillary contracture induced by an interwoven fibrous band in the 10-year-old girl.
